# Ten quick tips for delivering programming lessons

**DOI:** 10.1371/journal.pcbi.1007433

**Published:** 2019-10-31

**Authors:** Greg Wilson

**Affiliations:** RStudio, Inc., Toronto, Canada; University of Toronto, CANADA

## Abstract

Teaching well is a craft like any other, and success often comes from an accumulation of small improvements rather than from any single large change. This paper describes 10 practices you can use when teaching programming (and other subjects). All are easy to adopt and have proven their value in institutional classrooms, intensive workshops, and other settings.

## Introduction

Teaching well is a craft like any other, and success often comes from an accumulation of small improvements rather than from any single large change. This paper describes 10 practices you can use when teaching programming and other subjects that are easy to adopt and have proven their value in institutional classrooms and other settings, such as workshops. Some have been inspired by [1, 2, 3], while others draw on the experience of this paper's author and colleagues [4, 5, 6]. In particular, this paper extends the evidence-based practices described in [7].

The foundation for these recommendations is the fact that active learning is more effective than passive learning [8, 9]. People who use new knowledge as it comes in by doing exercises or summarizing it learn more than people who just watch or listen. Active teaching is similarly more effective: people learn more when instructors dynamically adjust their teaching based on real-time feedback from their learners, e.g., by providing an alternative explanation of a concept that the class has found difficult or by changing direction to incorporate a question that reveals an unexpected learner interest. Finally, learners who are intrinsically motivated learn more than those who are extrinsically motivated or not motivated at all [10]. When you make a connection between what you are teaching and your learners' goals or demonstrate that you respect their time and priorities, you increase how much they learn.

## Tip 1: Use formative assessment every 10–15 minutes

Instructors always want to get through more material than time allows, so we often teach at the speed at which we can talk rather than the speed at which people can learn. Having learners do something every 10–15 minutes slows us down to the speed at which people can learn rather than the speed at which we can talk. It also keeps them engaged and gives us and them feedback on whether they have actually understood.

In-class checks like this are called formative assessments. Good ones take only a minute or two to complete so that they don't derail the flow of the lesson and have an unambiguous correct answer so that they can be checked in large classes. Popular kinds of formative assessment in programming classes include:

- Answer a multiple choice question.
- Write a few lines of code.
- Predict what the code on the screen will do when it runs.
- Contribute the next line of code.
- Label a diagram of a data structure.
- Trace the order in which statements are evaluated.

Starting with a formative assessment that reviews a previous lesson is a good way to signal that class has started, and having learners recall older material before tackling something new improves learning outcomes [11]. Similarly, ending the class with such an exercise gives learners a sense of how far they have progressed.

Some formative assessments should be designed in advance; in fact, they should be designed before the lesson content is written so that they can act as goalposts [6]. However, they can also be created on the fly to incorporate and respond to learners' questions and confusions. For example, after writing and presenting a few lines of code, an instructor can ask what would happen if something was added or modified. If learners make different predictions, the instructor can then ask them to debate the outcomes as a form of ad hoc peer instruction [7].

## Tip 2: Give learners and yourself a break every 45–90 minutes

People's brains get tired when they are concentrating, and tired brains can't learn [9]. Caffeine doesn't fix this, so find an excuse, such as stand-up discussion, to have learners get up and move around for a few minutes every hour in order to reoxygenate their gray cells. This also allows those who need a bathroom break to take care of things discreetly. (A colleague once told me that the basic unit of teaching is the bladder. When I said I'd never thought of that, she said, "You've obviously never been pregnant.")

Hourly breaks aren't just for the learners' benefit. They also give instructors a few minutes to review what they are planning to teach next and to think about how to answer questions in their backlog (discussed in the next tip). If you are co-teaching (Tip 8), this is a natural time to swap roles, give or get feedback, or discuss problems that learners seem to be having.

## Tip 3: Use a variety of exercise types

The final rule in [7] said, "Don't just code," and it bears elaboration here. Most programming classes rely primarily on code-and-run exercises in which learners write software that behaves in a tightly specified way. To keep learners engaged and to give them opportunities to practice other skills and higher-level reasoning, you should also use the following:

**Parsons Problems**, which give them the lines of code needed to solve a problem but in jumbled order [12, 13, 14]. Parsons Problems reduce cognitive load by allowing learners to focus on control flow without simultaneously having to recall syntax.

**Multiple choice questions** whose wrong answers have been chosen to probe for specific misconceptions. For example, learners who have worked with spreadsheets may believe that after executing a = 10, b = a, and a = 20, the value of b will be 20.

**Matching and ranking problems** in which they match terms from one column to definitions in another, put predefined labels on a diagram, or sort items according to some criteria (e.g., most likely to least likely).

**Debugging, completion,** and **extension exercises** in which learners must fix, finish, or extend an existing program. These all model authentic tasks (i.e., the kinds of things programmers spend most of their time doing in real life).

**Tracing execution order** or **tracing values**, in which the learner lists the order in which the statements in a program are executed or the values that one or more variables take on as the program runs, which are essential program comprehension skills.

**Code reviews** in which learners score a program against a marking guide supplied by the instructor in order to learn how to find flaws in code. Learners start with a perfect score and lose points for false positives, as well as false negatives so that they don't simply mark every statement as being wrong in all possible ways.

## Tip 4: Use sticky notes to monitor progress and distribute attention

Sticky notes are my favorite teaching tool, and I'm not alone in loving their versatility, portability, stickability, foldability, and subtle yet alluring aroma [15]. Give each learner two sticky notes of different colors, such as orange and green. If someone has completed an exercise and wants it checked or if they feel that they are following the lesson, they put the green sticky note somewhere the instructor can see. If they run into a problem and need help, they put up the orange one. This works much better than having people raise their hands: it's more discreet (which means they're more likely to actually do it), they can keep working while their flag is raised rather than trying to type one-handed, and the instructor can quickly see from the front of the room what state the class is in.

Sticky notes can also be used to ensure that attention is fairly distributed. Instructors naturally focus their attention on learners who are making eye contact and asking lots of questions—in other words, on extroverts. This creates two feedback loops: the extroverts ask even more questions because they're getting attention, while other learners stop trying to engage because they aren’t. To prevent this, have each learner write their name on a sticky note and post it on their laptop or somewhere equally visible. Each time the instructor calls on them or answers one of their questions, they take their sticky note down. Once all the sticky notes are down, everyone puts theirs up again. This technique makes it easy for the instructor to see whom they haven't spoken with recently, which helps them avoid unconscious bias and preferential interaction. It also shows learners how attention is being distributed so that when they are called on, they won't feel like they're being picked on.

## Tip 5: Create a visible backlog

You may not have time to answer all of your learners' questions or might not actually know the answers. To handle this, write questions on sticky notes and post them on the wall behind you, then look over this backlog during breaks and decide which questions you want to tackle. This gives you a chance to prioritize based on what's most relevant (and what you actually know). It also helps build trust: Many people have learned that "I'll address that later" means "I hope you'll forget that you asked." If they see you trying to tackle a few of the questions that have come up, they will forgive you for not getting to the rest.

## Tip 6: Have learners take notes

Fifty years ago, when being able to summarize a speech or take minutes in a meeting was considered an essential white-collar skill, it was common for high school teachers to require students to hand in their notes for grading. This practice has fallen out of fashion, even though research shows that taking notes improves learning because it forces learners to organize and reflect on information as it's coming in, which in turn increases the likelihood that they will transfer it to long-term memory [16, 17].

To help learners improve their note-taking, have them take a minute at the end of each class to write one thing they learned on one side of a card and one question they still have or something they're confused about on the other. Reviewing these cards before the next class only takes a few minutes and will quickly reveal what learners have missed or misunderstood.

Another technique is to make 4–6 learners the official note takers for each class. They must summarize the information presented and find answers to backlog questions that the instructor didn't get to (Tip 5). Their notes are then graded by the instructor for quality and comprehensiveness and shared with the class (e.g., by being posted on the course website). In many cases, their notes will be more useful than what you might have put together, since they will record what they and their peers actually need to know rather than what you think they do.

## Tip 7: Present diagrams incrementally to complement other material

Our brains have separate channels for processing visual and linguistic information, so people learn best when complementary material is presented simultaneously through these channels [18, 19]. In simple terms, this means that you should present diagrams or other relevant images for you to talk about rather than slabs of text that duplicate what you are saying. Diagrams are even more effective if they are built up piece by piece rather than shown all at once. When this is done, learners' brains will correlate the arrival of new visual information with the arrival of new linguistic information. Presentation of either later on will then help trigger recall of the other.

All graphics should be directly relevant to the course material. For example, [20] distinguished between seductive graphics (which are highly interesting but not directly relevant to the instructional goal), decorative graphics (which are neutral but not directly relevant to the instructional goal), and instructive graphics (which are directly relevant to the instructional goal). Learners who received any kind of graphic gave material higher satisfaction ratings than those who didn't get graphics, but only learners who got instructive graphics actually performed better.

## Tip 8: Teach together

Co-teaching describes two instructors working together in the same classroom [21]:

**Team teaching:** The instructors take turns delivering content. Each can speak for the 10–15 minutes leading up to a formative assessment (Tip 1) or for the 45–90 minutes between breaks (Tip 2).

**Teach and assist:** Instructor A teaches while Instructor B moves around the classroom to help struggling learners. (This is often combined with team teaching: whoever isn't at the front of the class acts as a helper.)

**Alternative teaching:** Instructor A provides a small set of learners with more intensive or specialized instruction while Instructor B delivers a general lesson to the main group.

**Teach and observe:** Instructor A teaches while Instructor B observes the learners, collecting data on their understanding to help plan future lessons.

**Parallel teaching:** The class is divided in two, and the instructors present the same material simultaneously to each.

**Station teaching:** The learners are divided into small groups that rotate from one station or activity to the next while instructors supervise where needed.

Team teaching is particularly beneficial in day-long workshops because it gives each instructor a chance to rest and think about what they are going to do next. If you and a partner are co-teaching, try the following:

- Take 2–3 minutes before the start of each class to confirm who's teaching what.
- Work out a couple of hand signals as well. "You're going too fast," "Speak up," "That learner needs help," and "It's time for a bathroom break" are all useful.
- Each person should teach for at least 10–15 minutes at a time so that learners aren't distracted by frequent switch-ups. Hour-long turns that synchronize with breaks for learners are usually easiest to manage (see Tip 2).
- The person who isn't teaching shouldn't interrupt, offer corrections or elaborations, or do anything else to distract from what the person teaching is doing or saying but may ask leading questions if the learners seem lethargic or unsure of themselves.

Most importantly, take a few minutes when the class is over to congratulate or commiserate with each other: In teaching, as in life, shared misery is lessened and shared joy increased.

## Tip 9: Include everyone

As discussed in [6], inclusivity is a policy of including people who might otherwise be excluded or marginalized. [22] describes things instructors can do to make their lessons more accessible to learners with physical challenges, whereas [23] is a brief, practical guide to practices that will help everyone, not just members of marginalized groups:

**Ask learners to email you beforehand** to explain how they believe what they're about to learn will help them achieve their goals.

**Use inclusive language,** such as mixed or gender-neutral pronouns, culturally diverse names, etc.

**Avoid intimidating language,** e.g., the use of jargon or feigned surprise ("Oh, you don't know that?").

**Emphasize that what matters is the rate at which people learn,** not the advantages or disadvantages they had when they started.

## Tip 10: Enforce a code of conduct

The most important step in creating an inclusive classroom, and often the most difficult, is dealing with people who are being condescending or abusive. As a first step, adopt a code of conduct, tell everyone where to find it, and require everyone who takes part in your classes to abide by it. It can't stop people from being offensive any more than laws against theft stop people from stealing, but it can make expectations and consequences clear and signal that you are trying to make your class welcoming to all.

A code of conduct is only useful if it is enforced. If you believe that someone has violated yours, you may warn them, ask them to apologize, and/or expel them, depending on the severity of the violation, whether or not you believe it was intentional, and whether it is a repeated offense. If you do have to expel someone:

**Do it in front of witnesses.** Most people will tone down their language and hostility in front of an audience, and having someone else present ensures that later discussion doesn't degenerate into conflicting claims about who said what.

**Tell the rest of the class what happened and why.** This helps prevent rumors from spreading and shows that your code of conduct actually means something.

**Email the offender as soon as you can** to summarize what happened and the steps you took and copy the message to your workshop's hosts or one of your fellow teachers so that there's a contemporaneous record of the conversation. If the offender replies, don't engage in a long debate: it's never productive.

What happens outside of class matters at least as much as what happens within it [24], so you need to provide a way for learners to report problems that you aren't there to see yourself. One step is to ask someone who isn't part of your group to be the first point of contact; that way, if someone wants to make a complaint about you or one of your fellow teachers, they have some assurance of confidentiality and independent action. [25] has lots of other advice and is both short and practical.

## Conclusion

One final note: If you are teaching an evening class after working for a full day, you and your learners will both appreciate it if you brush your teeth and put on a clean shirt before you start teaching. Cough drops will also help you keep your voice and fend off whatever colds the learners brought with them, and your back will be grateful tomorrow that you wore comfortable shoes today.
